# A Carbonaceous Membrane based on a Polymer of Intrinsic Microporosity (PIM-1) for Water Treatment

**DOI:** 10.1038/srep36078

**Published:** 2016-10-26

**Authors:** Hee Joong Kim, Dong-Gyun Kim, Kyuchul Lee, Youngbin Baek, Youngjae Yoo, Yong Seok Kim, Byoung Gak Kim, Jong-Chan Lee

**Affiliations:** 1 School of Chemical and Biological Engineering and Institute of Chemical Processes, Seoul National University, 599 Gwanak-ro, Gwanak-gu, Seoul 08826, Republic of Korea; 2 Advanced Materials Division, Korea Research Institute of Chemical Technology, 141 Gajeongro, Yuseong-gu, Daejeon 34114, Republic of Korea; 3 Department of Chemical Convergence Materials, University of Science and Technology, 217 Gajeongro, Yuseong-gu, Daejeon 34114, Republic of Korea

## Abstract

As insufficient access to clean water is expected to become worse in the near future, water purification is becoming increasingly important. Membrane filtration is the most promising technologies to produce clean water from contaminated water. Although there have been many studies to prepare highly water-permeable carbon-based membranes by utilizing frictionless water flow inside the carbonaceous pores, the carbon-based membranes still suffer from several issues, such as high cost and complicated fabrication as well as relatively low salt rejection. Here, we report for the first time the use of microporous carbonaceous membranes via controlled carbonization of polymer membranes with uniform microporosity for high-flux nanofiltration. Further enhancement of membrane performance is observed by O_2_ plasma treatment. The optimized membrane exhibits high water flux (13.30 LMH Bar^−1^) and good MgSO_4_ rejection (77.38%) as well as antifouling properties. This study provides insight into the design of microporous carbonaceous membranes for water purification.

Carbon-based membranes have been extensively studied because of their unique characteristics, such as high physicochemical stability, fast mass transport behavior, large surface area, biocidal property, and narrow pore size distribution^1^,^2^,^3^,^4^,^5^,^6^,^7^,^8^,^9^,^10^,^11^. Based on these properties, they have been utilized in diverse applications including gas or liquid separation^1^,^2^,^3^,^4^, catalytic reactions^5^, chemical sensing^6^,^7^,^8^, energy storage^9^, and tissue engineering^10^. In particular, water treatment membranes consisting of carbon nanomaterials, such as carbon nanotube (CNT) and graphene derivatives, have a unique advantage of fast water permeation by the low frictional water flow through their carbonaceous pores^12^,^13^,^14^,^15^,^16^,^17^,^18^,^19^. For example, CNT array membranes with aligned 1D carbonaceous nanochannels exhibit ultrahigh water flux values, which are several orders of magnitude higher than those exhibited by conventional ultrafiltration (UF) membranes^13^,^14^,^15^,^16^. Graphene oxides (GO) membranes with 2D carbonaceous nanochannels have been reported to exhibit fast water flux with controlled separation performance for sub-10 nm particles and molecules^17^,^18^,^20^. However, practical applications of these membranes are still limited by several issues, such as high cost and complicated fabrication for CNT-based membranes^13^,^14^,^15^,^16^,^17^, as well as poor stability under hydrated conditions and difficult pore size control for graphene-based membranes^4^,^20^. In addition, both membranes often suffer from relatively low salt rejection rates, attributed to the large pore size of CNT-based membranes^13^ and the deterioration of integrity of graphene-based membranes by the hydration^4^,^20^, which in turn hampers the application of the membranes for nanofiltration (NF) or reverse osmosis (RO). Hence, a more convenient and efficient method for preparing carbonaceous membranes with a high flux and salt rejection rate is required for the water treatment applications.

Microporous polymers are of great interest as promising next-generation molecular sieving and storage materials for the applications of gas sorption, separation and storage, pervaporation, and catalytic supports^21^,^22^,^23^,^24^,^25^. Recently, polymers of intrinsic microporosity (PIMs), a novel class of microporous polymers, have attracted considerable attention because of good solubility and processability, different available functional groups, high glass transition temperature, good thermal stability, and excellent mechanical and film-forming properties^26^,^27^,^28^,^29^,^30^,^31^,^32^,^33^,^34^. As PIMs contain fused-ring and ladder-like structures integrated with contortion sites, they have uniform interconnected micropores (<2 nm) and a high surface area (300–1000 m^2^ g^−1^)^30^,^31^,^32^. Several studies have reported the use of PIM membranes for gas separation by exploiting their high gas permeability and selectivity^26^,^27^,^28^,^30^,^31^,^33^,^34^; however, only a few studies have reported the use of PIM membranes for the filtration of organic solutions^32^. Moreover, thus far, a PIM membrane for water treatment applications has not been reported because it is difficult to utilize the hydrophobic micropores of PIMs for transporting water molecules. Considering the low frictional water flow through the pores of carbonaceous membrane, it might be possible to prepare microporous, carbonized PIM membranes with high water flux and selectivity by carbonization of the PIM membranes.

Previously, we have reported the preparation of 2–15 nm thick, graphene-like carbonaceous thin films on a quartz substrate by the carbonization of thin films of a polymer of intrinsic microporosity (PIM-1)^35^. Herein, we report the fabrication of a new type of free-standing carbonaceous membrane based on PIM-1 via controlled carbonization; this membrane exhibits interconnected, sub-1 nm pores with a narrow size distribution. These characteristics result in high flux and a good salt rejection rate for the filtration of an MgSO_4_ aqueous solution, thus making the membrane attractive for NF applications. In addition, the water flux and antifouling property of the membrane can be further enhanced without sacrificing the salt rejection rate by subjecting the membrane to O_2_ plasma treatment.

## Results

**Figure 1:**
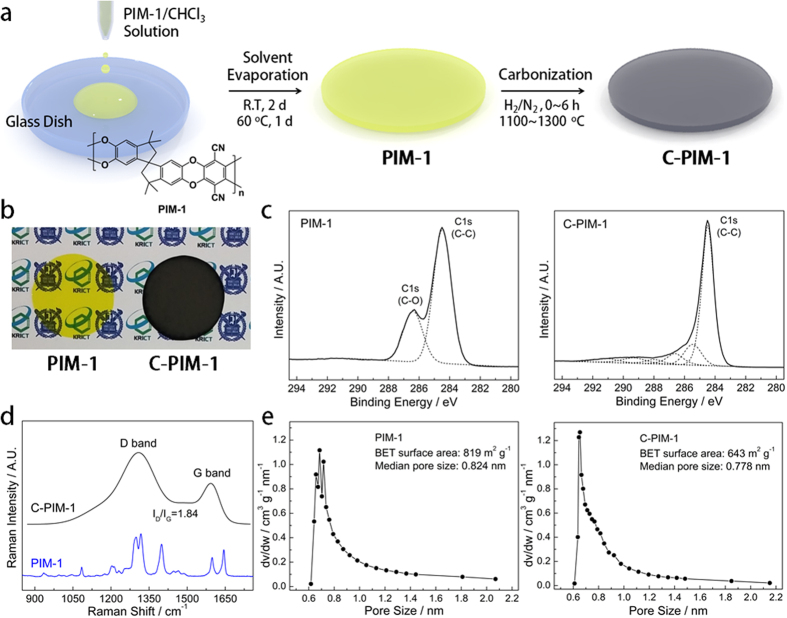
Preparation and characteristics of PIM-1 and C-PIM-1 membranes. **(a**) Preparation procedure of PIM-1 and C-PIM-1 membranes. (**b**) Photographs of the PIM-1 and C-PIM-1 membranes. (**c**) XPS C 1s spectra, (**d**) Raman spectra, and (**e**) pore size distributions of the PIM-1 and C-PIM-1 (40% carbonization) membranes.

PIM-1 was synthesized by polycondensation of 5,5′,6,6′-tetrahydroxy-3,3,3′,3′-tetramethyl-1,1′-spirobisindane (TTSBI) and 2,3,5,6-tetrafluoroterephthalonitrile (TFTPN), as previously reported^29^,^35^,^36^,^37^. ^1^H NMR and elemental analysis (EA) revealed that the polymer was successfully synthesized (see Methods, Supplementary Information). The number-average molecular weight (*M*_n_) and molecular weight distribution (*Đ*) of PIM-1, obtained by gel-permeation chromatography (GPC), are 50,100 g mol^−1^ and 1.87, respectively. A PIM-1 membrane was prepared by a simple solution casting method (Fig. 1a); a solution of PIM-1 in CHCl_3_ was poured into a glass dish (diameter = 10 cm), followed by the slow evaporation of the solvent at room temperature. The thickness of the PIM-1 membrane was controlled by changing the concentration (0.5–2.0 wt%) and amount of the PIM-1 casting solution. After the PIM-1 membrane was completely dried under vacuum at 60 °C, controlled thermal treatment under N_2_/H_2_ atmosphere (95/5 vol%) was conducted to fabricate a carbonaceous PIM-1 membrane (C-PIM-1). The yellow transparent PIM-1 membrane changed into a glittering-grey opaque C-PIM-1 membrane after carbonization (Fig. 1b; Fig. S1, Supplementary Information). The degree of carbonization, defined as the membrane weight loss (%) during thermal treatment, was controlled by changing the temperature (1,100–1,300 °C) and time (1–6 h). As shown in Table S1 (Supplementary Information), the degree of carbonization for the C-PIM-1 membranes was controlled from 37.5% to 60%. Unfortunately, it was difficult to prepare C-PIM-1 membranes with a degree of carbonization below ≈35% due to the abrupt weight loss of PIM-1 from 0% to ≈35%. The abrupt weight loss of PIM-1 could be also observed by TGA under N_2_ flow (Fig. S2, Supplementary Information), although the actual decomposition temperature under N_2_/H_2_ flow (95/5 vol%) might be different from the TGA result. In addition, the C-PIM-1 membranes with a degree of carbonization higher than 60% were prepared, however, they were too fragile to be used as the pressure-driven filtration membranes. Thus, C-PIM-1 membranes with a degree of carbonization from 37.5% to 60% were used because they are sufficiently robust, maintaining their free-standing film state from the filtration even under an applied pressure of 10 bar.

The carbonization the PIM-1 membrane to the C-PIM-1 membrane via the thermal treatment could be monitored by X-ray photoelectron spectroscopy (XPS) analysis; the carbon content of the membrane increases from 82.60 at% to 96.82 at% upon the carbonization process, while the content of oxygen and nitrogen decreases (Table S2, Supplementary Information). In addition, the content of carbon in the C–C bond (284.4 eV) of the C-PIM-1 membrane was found to be much larger than that of the PIM-1 membrane (Fig. 1c). The atomic composition results, obtained from EA and XPS experiments, indicate the uniform carbonization from surface to inside part of the membrane (Table S2, Supplementary Information). Raman spectroscopy clearly shows the D (1310 cm^−1^) and G (1595 cm^−1^) band peaks, corresponding to the graphitic carbon structures of the C-PIM-1 membrane (Fig. 1d)^38^,^39^,^40^, while such graphitic carbon structural peaks were not observed for the PIM-1 membrane. In addition, the relative intensity of D3 peak at 1500 cm^−1^, compared to that of G peak at 1595 cm^−1^, decreases with increasing the degree of carbonization; D3 and G peaks correspond to amorphous carbon and graphitic carbon lattice, respectively (Fig. S3, Supplementary Information). Therefore, C-PIM-1 membrane with a high degree of carbonization has low amorphous carbon content^38^,^39^. The degree of crystallinity, calculated from the integrated intensity ratio of the D and G bands (*I*_D_/*I*_G_), is 1.84 for the C-PIM-1 membrane with 40% carbonization; this is typical value for the carbonaceous materials prepared by the thermal treatment of polymer precursors^41^,^42^. The change of surface morphology of the membranes could be observed from scanning electron microscopy (SEM) and atomic force microscopy (AFM) analyses; a quite flat surface (root-mean-square roughness, *R*_q_ = 0.85 ± 0.26) of the PIM-1 membrane was found to be changed to a relatively rough surface (*R*_q_ = 15.51 ± 2.10) for the C-PIM-1 membrane, attributed to the nanoscale thermal shrinkage by the carbonization (Figs S4 and S5, Supplementary Information)^43^,^44^. Still the interconnected micropore characteristics of the PIM-1 membrane having median pore size of 0.824 nm and surface area of 819 m^2^ g^−1^ are preserved for some degree after the carbonization for the C-PIM-1 membrane having median pore size of 0.778 nm and surface area of 643 m^2^ g^−1^ (Figs 1e and S6, Supplementary Information).

**Figure 2:**
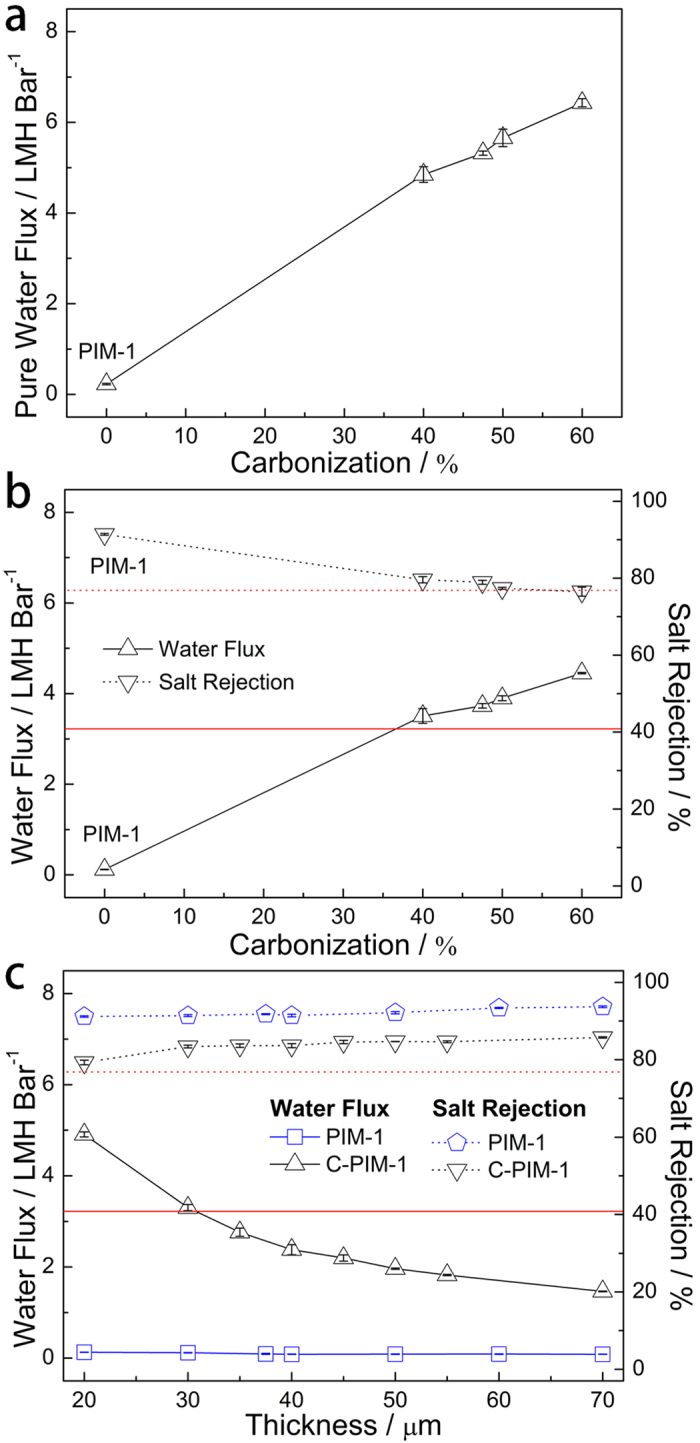
Water flux and salt rejection performance of the PIM-1 and C-PIM-1 membranes. The effect of degree of carbonization on water flux and salt rejection of the membranes with a thickness of 30 μm upon (**a**) pure water and (**b**) MgSO_4_ solution (2,000 ppm) filtrations. (**c**) The effect of membrane thickness on water flux and salt rejection of the membranes with the degree of carbonization of 37.5% upon MgSO_4_ solution (2,000 ppm) filtration. The red solid and dotted lines indicate the water flux and salt rejection of a commercial polyamide NF membrane (NF2A), respectively.

Dead-end filtration test was performed to evaluate the pure water permeability behavior of the C-PIM-1 membrane with a thickness of 30 μm. Figure 2a clearly shows the very large increase of the pure water flux after the carbonization; pure water flux increases from 0.23 LMH bar^−1^ for the PIM-1 membrane to 6.43 LMH bar^−1^ for the C-PIM-1 membrane with 60% carbonization, which is a 28-fold increase in the water flux as a result of carbonization. The increase in water flux by carbonization is attributed to the low frictional water flow inside the carbonaceous pores rather than the pore size and surface area of the membranes^12^,^13^,^14^,^15^,^16^,^17^,^18^,^19^. A solution-diffusion model, which is widely used to explain mass transport through dense membranes with sub-1 nm pores, was employed in order to elucidate the increase of water permeability by the carbonization^12^,^45^,^46^. Water flux (*J*_w_, g cm^−2^ s^−1^) in the solution-diffusion model is expressed as follows:


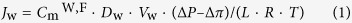


where *C*_m_^W,F^ is the equilibrium water concentration in the membrane (g H_2_O in a 1 cm^−3^ swollen membrane), *D*_w_ is the average water diffusion coefficient in the membrane (cm^2^ s^−1^), *V*_w_ is the partial molar volume of water (18.0 cm^3^ mol^−1^), which is typically approximated by the molar volume of pure water^45^,^46^, Δ*P* is the difference in pressure between feed and permeate (bar), Δ*π* is the osmotic pressure difference across the membrane (bar), *L* is the membrane thickness (cm), *R* is the gas constant (83.1 cm^3^ bar mol^−1^ K^−1^), and *T* is the absolute temperature (298 K). Two parameters, *C*_m_^W,F^ and *D*_w_, should be the key factors in determining the water flux behavior for the PIM-1 and C-PIM-1 membranes because all the other parameters are identical. The *C*_m_^W,F^ of the membranes was evaluated by the measurement of the equilibrium water uptake of the membranes in pure water (Fig. S7, Supplementary Information). The *C*_m_^W,F^ of the C-PIM-1 membrane with 40% carbonization (5.52 × 10^−2^ g H_2_O in a 1 cm^3^ swollen membrane) is approximately 4.7 times larger than that of the PIM-1 membrane (1.18 × 10^−2^ g H_2_O in a 1 cm^3^ swollen membrane). *C*_m_^W,F^ was also found to increase with the degree of carbonization. A membrane with a large *C*_m_^W,F^ is known to exhibit high water permeability because the larger amount of water in the membrane pores can provide the pathways for water molecules (i.e., convective frame of reference effect)^45^,^46^. The calculated *D*_w_ values of the C-PIM-1 membranes (7.08 × 10^−3^–8.90 × 10^−3^ cm^2^ s^−1^) are approximately 4.8–6.1 times larger than that of the PIM-1 membrane (1.47 × 10^–3^ cm^2^ s^−1^), which are close to those of other carbon-based membranes (5 × 10^−3^–8 × 10^−3^ cm^2^ s^−1^)^47^,^48^. Those of conventional polymeric membranes are in the range of 1 × 10^−4^ to 1 × 10^−7^ cm^2^ s^−1 ^45,46. Therefore, the water diffusion behavior of the C-PIM-1 membrane is similar to that in the carbon-based membranes. The carbon-based membranes containing CNT and graphene derivatives have well-defined micropores and exhibit low frictional water flow inside the carbonaceous pores via the formation of agglomerated hydrogen bonds between water molecules, thus resulting in the high water permeability^47^,^48^. The much larger *C*_m_^W,F^ and *D*_w_ values of the C-PIM-1 membrane than those of the PIM-1 membrane can be explained for some degree by water contact angle study (Fig. S8, Supplementary Information). It is well known that membranes with high water wettability exhibit large water sorption and diffusion coefficients^45^,^46^. The C-PIM-1 membrane shows smaller water contact angle and higher water wettability than PIM-1 membrane possibly due to its graphitic carbon structure^49^ and rough surface morphology^50^,^51^, as presented in the Raman spectroscopy and AFM results, respectively (Figs 1d and S5, Supplementary Information). It has been reported that clean graphene surface exhibited quite low water contact angle value (37 °), °riginated from the strong interaction between graphene surface and water molecules^49^. Higher water wettability of membrane could also be obtained by introducing the rough surface morphologies^50^,^51^.

Subsequently, the NF performance of the C-PIM-1 membrane was investigated using an aqueous MgSO_4_ solution. The pure water flux behavior of the C-PIM-1 membrane is mirrored in Fig. 2b for the MgSO_4_ solution filtration, where the C-PIM-1 membrane also shows an increase of water flux with increasing degree of carbonization, and exhibits much larger water flux (3.51–4.45 LMH bar^−1^) than the PIM-1 membrane (0.12 LMH bar^−1^). Although the salt rejection rates of the C-PIM-1 membranes (78.76–82.94%) are somewhat smaller than that of PIM-1 membrane (91.41%) due to the typical trade-off behavior between water diffusion coefficient and water/salt selectivity^45^,^46^, those are still comparable to or slightly larger than that of a commercial polyamide (PA) NF membrane (NF2A) (76.86%) measured in this study. The NF performance of NF2A is worse than that in the technical specification provided by the company, however, such discrepancy has been also reported by others, which is attributed to the effect of the membrane filtration condition^52^. The high salt rejection rate of the high-flux C-PIM-1 membrane is consistent with the BET results, which demonstrate the sub-1 nm sized, interconnected carbonaceous pores present in the membrane (Figs 1e and S6, Supplementary Information). Figure 2c shows that the C-PIM-1 membranes as thin as 20 μm can be easily prepared, yielding water flux as high as 4.91 LMH bar^–1^ for the MgSO_4_ solution filtration, when the degree of carbonization of the membrane is 37.5%. The increase in the water flux of the C-PIM-1 membrane with decreasing membrane thickness is attributed to the reduction of thickness resistance (Equation (1))^17^,^46^. The salt rejection rate is almost independent of the membrane thickness, indicating that membranes are substantially free from micro- or several nanometer-scale defects. 20 μm was found to be the minimum thickness for the free-standing C-PIM-1 membrane to have the physical and mechanical stability under the high pressure of NF. The water flux behavior of PIM-1 membranes with different thicknesses is similar to that of C-PIM-1 membrane (Table S1, Supplementary Information). However, because of their small values, the changes in water flux of the PIM-1 membrane were not clearly seen in Fig. 2c.

**Figure 3:**
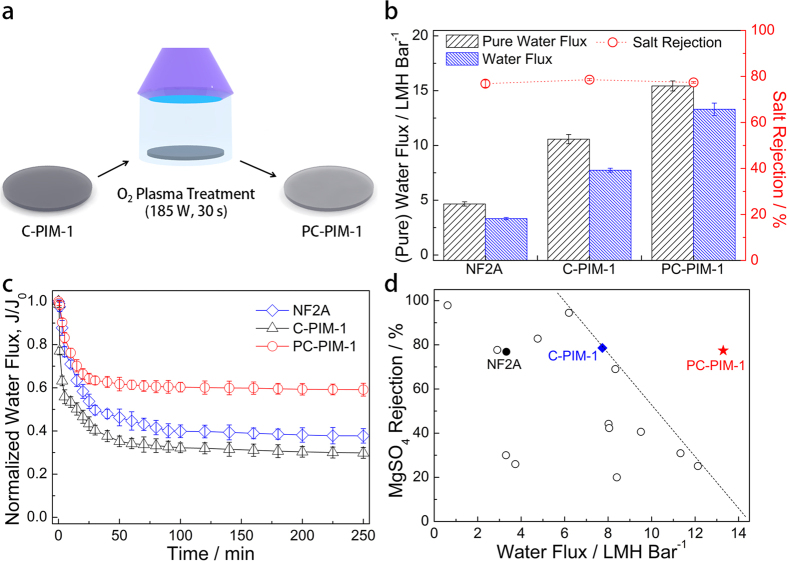
Preparation and performance of O_2_ plasma-treated C-PIM-1 membrane (PC-PIM-1). (**a**) Preparation procedure of PC-PIM-1 membrane. (**b**) Pure water flux, water flux, and salt rejection performance of NF2A, and C-PIM-1 and PC-PIM-1 membranes with a thickness of 20 μm and a degree of carbonization of 60%. (**c**) Time-dependent normalized water flux variations of the NF2A, C-PIM-1, and PC-PIM-1 (20 μm, 60% carbonization) membranes during BSA solution (1 g L^−1^) filtration. (**d**) MgSO_4_ rejection rate and water flux performance of optimized C-PIM-1 and PC-PIM-1 membranes in this study and other NF membranes in the literature.

Carbon-based membranes, such as CNT array membranes, are known to exhibit a large entrance/exit resistance for water molecules to pass through the inner pores of the membranes^13^,^53^. For example, the entrance and exit resistances are larger than 120 bar and 1,000 bar, respectively, for the CNT array membrane, calculated by the molecular dynamic simulations^13^,^53^. As compared to the CNT array membranes, the C-PIM-1 membrane possibly exhibits a relatively smaller entrance/exit resistance^13^,^53^, as expected from its better water wettability (Fig. S8, Supplementary Information). Still, the water permeability of the C-PIM-1 membrane can be further improved by hydrophilic surface modification for decreasing the entrance/exit resistance. Both surfaces of the C-PIM-1 membrane were subjected to O_2_ plasma for preparing the O_2_ plasma-treated C-PIM-1 membrane (PC-PIM-1), as illustrated in Fig. 3a. The oxygen content on the membrane surface, analyzed by XPS, significantly increases by the O_2_ plasma treatment (Table S2, Supplementary Information), thereby increasing the water wettability on the membrane surface (Fig. S8, Supplementary Information), while the bulk atomic composition of the membrane does not change much as observed from EA measurement. This clearly demonstrates that hydrophilic oxygen functional groups are formed on the membrane surface by the O_2_ plasma treatment without changing the inner carbonaceous structure of the membrane. Furthermore, *I*_D_/*I*_G_ ratios of C-PIM-1 and PC-PIM-1 membranes were found to be close from the Raman spectroscopy, indicating that the graphitic carbon structures on the C-PIM membrane are not damaged during O_2_ plasma treatment (Fig. S9, Supplementary Information). The effect of O_2_ plasma treatment on membrane surface morphologies was also investigated by SEM and AFM (Figs S10 and S11, Supplementary Information); any distinct change was not observed after the O_2_ plasma treatment, indicating that the O_2_ plasma treatment does not change the surface morphologies much. The hydrophilic functional groups imparted by the O_2_ plasma treatment were found to stably remain even after exposed to air for a week. The overall water permeability behavior of the PC-PIM-1 membranes with different degrees of carbonization and thicknesses is close to that of the C-PIM-1 membranes, while the water permeability of the PC-PIM-1 membranes is about 1.5 times higher than that of the C-PIM membranes due to the decreased entrance/exit resistance (Table S1, Supplementary Information). We could obtain the highest water flux from a PC-PIM-1 membrane with a thickness of 20 μm and 60% carbonization; 15.43 LMH bar^–1^ and 13.30 LMH bar^−1^ for the filtration of pure water and MgSO_4_ solution, respectively, as shown in Fig. 3b.

The salt rejection rate is generally assumed to decrease with increasing water flux of filtration membranes^45^,^46^. However, both C-PIM-1 and PC-PIM-1 membranes exhibit similar salt rejection performance, despite the significant increase in water flux for the membranes after O_2_ plasma treatment (Fig. 3b; Table S1, Supplementary Information). This result could be attributed to the presence of negatively charged oxygen functional groups on the PC-PIM-1 membrane (Fig. S12, Supplementary Information), which can improve the salt rejection rate by electrostatic repulsion (i.e., Donnan exclusion ability)^54^,^55^. To investigate the Donnan exclusion ability of the PC-PIM-1 membrane, filtration experiments were conducted with various salt solutions having different ion valences under a relatively low feed pressure (5 bar) and low salt concentration (10 mM) for minimizing the transport of ions by convection and diffusion, respectively (Fig. S13, Supplementary Information)^54^,^55^. Considering the hydrated salt size and charge effects, the rejection (*R*) of salt solutions should follow the orders of *R*(MgSO_4_) > *R*(MgCl_2_) > *R*(Na_2_SO_4_) > *R*(NaCl) and *R*(Na_2_SO_4_) > *R*(MgSO_4_) ≈ *R*(NaCl) > *R*(MgCl_2_), respectively (Table S3, Supplementary Information)^17^,^54^. The rejection of salt solutions of the C-PIM-1 membrane follows the order of *R*(MgSO_4_) > *R*(MgCl_2_) > *R*(Na_2_SO_4_) > *R*(NaCl), indicating that the salt rejection of the C-PIM-1 membrane is mainly determined by the size effect. However, the rejection of the PC-PIM-1 membrane follows the order of *R*(MgSO_4_) > *R*(Na_2_SO_4_) > *R*(MgCl_2_) > *R*(NaCl); the change of the rejection order and the significant increase for *R*(Na_2_SO_4_) and *R*(NaCl) are observed for the PC-PIM-1 membrane, demonstrating that the salt rejection of the PC-PIM-1 membrane is determined by both of charge and size. Therefore, the PC-PIM-1 membrane shows increased water flux without decreasing the salt rejection compared to the C-PIM-1 membrane due to the Donnan exclusion from the negatively charged surface functional groups.

Antifouling properties of the membranes were also evaluated using bovine serum albumin (BSA) as a model foulant, which is the most commonly used protein foulant for the antifouling tests^56^,^57^,^58^,^59^,^60^,^61^,^62^,^63^. Figure 3c presents the time-dependent normalized water flux variations of the NF2A, C-PIM-1, and PC-PIM-1 membranes during the filtration of a BSA solution. The NF2A and C-PIM-1 membranes show larger flux decreases as compared to the PC-PIM-1 membrane, especially in the initial filtration stage. Upon reaching a steady state after 250 min of filtration, the flux decline ratio (DR) of the PC-PIM-1 membrane (40.8%) is much smaller than those of NF2A (62.3%) and C-PIM-1 (70.1%) membranes; interestingly, the C-PIM-1 membrane shows the largest DR possibly due to its non-polar and uncharged surface (Fig. S12, Supplementary Information)^62^,^63^. Thus, the treatment of the C-PIM-1 membrane by O_2_ plasma further imparts antifouling properties to the membrane against BSA, which would be another advantage of the O_2_ plasma treatment. A hydrophilic and charged membrane surface can provide an energetic barrier for the adhesion of foulants on the membrane surface via favorable water-surface interaction and electrostatic repulsion between foulants and the surface^60^,^62^,^63^.

Figure 3d displays the salt rejection and water fluxes of various NF membranes for the filtration of MgSO_4_ aqueous solutions (Table S4, Supplementary Information, for details). Most of the membranes reported previously have been found to exhibit a typical trade-off phenomenon. For example, a PA membrane exhibits a high salt rejection rate (94.5%) but low water flux (6.20 LMH bar^−1^) for the filtration of a 3,000 ppm MgSO_4_ solution^64^. In contrast, a graphene/CNT composite membrane shows the highest water flux (12.13 LMH bar^−1^) but a poor salt rejection rate (25.1%) for the filtration of a 1,200 ppm MgSO_4_ solution^19^. As compared with representative results across recently published studies, the C-PIM-1 membrane exhibits a comparable water flux and salt rejection rate. Furthermore, the high flux and good salt rejection rate of the PC-PIM-1 membrane clearly exceed the upper limit of state-of-the-art NF membrane performance. Although the reported MgSO_4_ rejection rate and water flux data were obtained under different conditions (Table S4, Supplementary Information), at least, such a comparison has demonstrated that the carbonaceous PIM-1 membrane with an O_2_ plasma-treated surface (PC-PIM-1) could act as a high-performance NF membrane.

## Discussion

We have demonstrated that a carbonaceous NF membrane (C-PIM-1) can be prepared by the controlled carbonization of a PIM-1 membrane. Sub-1 nm-sized, interconnected, low frictional carbonaceous pores of the C-PIM-1 membrane facilitate the permeation of water molecules through the membrane, leading to a high water flux and good salt rejection rate. Moreover, the O_2_ plasma treatment of the C-PIM-1 membrane results in water flux enhancement without decreasing the salt rejection rate, as well as high fouling resistance against proteins. These properties are attributed to the negatively charged hydrophilic membrane surface that decreases the entrance/exit resistance of the carbonaceous pores while facilitating the Donnan exclusion and reduces the interaction of proteins with the membrane surface. This study provides insight into the design and preparation of carbonaceous PIM membranes for versatile applications including the filtration. In particular, the modification of the chemical structure of PIMs can possibly control the pore characteristics of the corresponding carbonaceous PIM membranes. Currently, studies for the further improvement of these membranes, such as fabrication of a thin, selective layer of carbonized PIMs on a supporting membrane for increasing water flux, are underway in our laboratory.

## Methods

Materials and methods including membrane preparation details are described in the Supplementary Information.

## Additional Information

**How to cite this article**: Kim, H. J. *et al*. A Carbonaceous Membrane based on a Polymer of Intrinsic Microporosity (PIM-1) for Water Treatment. *Sci. Rep.* **6**, 36078; doi: 10.1038/srep36078 (2016).

**Publisher’s note:** Springer Nature remains neutral with regard to jurisdictional claims in published maps and institutional affiliations.

## Supplementary Material

Supplementary Information

## References

[b1] Karan S., Samitsu S., Peng X. S., Kurashima K. & Ichinose I. Ultrafast viscous permeation of organic solvents through diamond-like carbon nanosheets. Science 335, 444–447 (2012). 22282807 10.1126/science.1212101

[b2] Jiang D.-E., Cooper V. R. & Dai S. Porous graphene as the ultimate membrane for gas separation. Nano Lett. 9, 4019–4024 (2009). 19995080 10.1021/nl9021946

[b3] Huang L., Li Y. R., Zhou Q. Q., Yuan W. J. & Shi G. Q. Graphene oxide membranes with tunable semipermeability in organic solvents. Adv. Mater. 27, 3797–3802 (2015). 25994919 10.1002/adma.201500975

[b4] Hung W.-S. . Cross-linking with diamine monomers to prepare composite graphene oxide-framework membranes with varying d-spacing. Chem. Mater. 26, 2983–2990 (2014).

[b5] Lv R. T. . Open-ended, N-doped carbon nanotube-graphene hybrid nanostructures as high-performance catalyst support. Adv. Funct. Mater. 21, 999–1006 (2011).

[b6] Salehi-Khojin A. . On the sensing mechanism in carbon nanotube chemiresistors. ACS Nano 5, 153–158 (2011). 21186822 10.1021/nn101995f

[b7] Liu Y. X., Dong X. C. & Chen P. Biological and chemical sensors based on graphene materials. Chem. Soc. Rev. 41, 2283–2307 (2012). 22143223 10.1039/c1cs15270j

[b8] Sippel-Oakley J. . Carbon nanotube films for room temperature hydrogen sensing. Nanotechnology 16, 2218–2221 (2005). 20817998 10.1088/0957-4484/16/10/040

[b9] Che G. L., Lakshmi B. B., Fisher E. R. & Martin C. R. Carbon nanotubule membranes for electrochemical energy storage and production. Nature 393, 346–349 (1998).

[b10] Harrison B. S. & Atala A. Carbon nanotube applications for tissue engineering. Biomaterials 28, 344–353 (2007). 16934866 10.1016/j.biomaterials.2006.07.044

[b11] Liu S. B. . Antibacterial activity of graphite, graphite oxide, graphene oxide, and reduced graphene oxide: membrane and oxidative stress. ACS Nano 5, 6971–6980 (2011). 21851105 10.1021/nn202451x

[b12] Paul D. R. Creating new types of carbon-nased membranes. Science 335, 413–414 (2012). 22282798 10.1126/science.1216923

[b13] Lee B. . A carbon nanotube wall membrane for water treatment. Nat. Commun. 6, 7109 (2015). 25971895 10.1038/ncomms8109

[b14] Hinds B. J. . Aligned multiwalled carbon nanotube membranes. Science 303, 62–65 (2004). 14645855 10.1126/science.1092048

[b15] Holt J. K. . Fast mass transport through sub-2-nanometer carbon nanotubes. Science 312, 1034–1037 (2006). 16709781 10.1126/science.1126298

[b16] Sholl D. S. & Johnson J. K. Making high-flux membranes with carbon nanotubes. Science 312, 1003–1004 (2006). 16709770 10.1126/science.1127261

[b17] Han Y., Xu Z. & Gao C. Ultrathin graphene nanofiltration membrane for water purification. Adv. Funct. Mater. 23, 3693–3700 (2013).

[b18] Huang H. B. . Ultrafast viscous water flow through nanostrand-channelled graphene oxide membranes. Nat. Commun. 4, 2979 (2013). 24352165 10.1038/ncomms3979

[b19] Han Y., Jiang Y. Q. & Gao C. High-flux graphene oxide nanofiltration membrane intercalated by carbon nanotubes. ACS Appl. Mater. Interfaces 7, 8147–8155 (2015). 25837883 10.1021/acsami.5b00986

[b20] Yeh C.-N., Raidongia K., Shao J. J., Yang Q.–H. & Huang J. X. On the origin of the stability of graphene oxide membranes in water. Nat. Chem. 7, 166–170 (2015). 10.1038/nchem.2145 25615671

[b21] Ghanem B. S., Swaidan R., Ma X. H., Litwiller E. & Pinnau I. Energy-efficient hydrogen separation by ab-type ladder-polymer molecular sieves. Adv. Mater. 26, 6696–6700 (2014). 25043652 10.1002/adma.201401328

[b22] Zhang P. F., Li H. Y., Veith G. M. & Dai S. Soluble porous coordination polymers by mechanochemistry: from metal-containing films/membranes to active catalysts for aerobic oxidation. Adv. Mater. 27, 234–239 (2015). 25389070 10.1002/adma.201403299

[b23] Pandey P. . Imine-linked microporous polymer organic frameworks. Chem. Mater. 22, 4974–4979 (2010).

[b24] Carta M. . An efficient polymer molecular sieve for membrane gas separations. Science 339, 303–307 (2013). 23329042 10.1126/science.1228032

[b25] Guiver M. D. & Lee Y. M. Polymer rigidity improves microporous membranes. Science 339, 284–285 (2013). 23329040 10.1126/science.1232714

[b26] Bezzu C. G. . A spirobifluorene-based polymer of intrinsic microporosity with improved performance for gas separation. Adv. Mater. 24, 5930–5933 (2012). 22961917 10.1002/adma.201202393

[b27] Lau C. H. . Gas-separation membranes loaded with porous aromatic frameworks that improve with age. Angew. Chem. Int. Edit. 54, 2669–2673 (2015). 10.1002/anie.201410684 25586722

[b28] Song Q. L. . Controlled thermal oxidative crosslinking of polymers of intrinsic microporosity towards tunable molecular sieve membranes. Nat. Commun. 5, 4813 (2014). 25186051 10.1038/ncomms5813

[b29] Budd P. M. . Solution-processed, organophilic membrane derived from a polymer of intrinsic microporosity. Adv. Mater. 16, 456–459 (2004).

[b30] McKeown N. B. & Budd P. M. Polymers of intrinsic microporosity (PIMs): organic materials for membrane separations, heterogeneous catalysis and hydrogen storage. Chem. Soc. Rev. 35, 675–683 (2006). 16862268 10.1039/b600349d

[b31] Du N. Y. Polymer nanosieve membranes for CO_2_-capture applications. Nat. Mater. 10, 372–375 (2011). 21460822 10.1038/nmat2989

[b32] Gorgojo P. . Ultrathin polymer films with intrinsic microporosity: anomalous solvent permeation and high flux membranes. Adv. Funct. Mater. 24, 4729–4737 (2014).

[b33] Yong W. F. . Molecular engineering of PIM-1/Matrimid blend membranes for gas separation. J. Membr. Sci. 407–408, 47–57 (2012).

[b34] Yong W. F., Kwek K. H. A., Liao K.-S. & Chung T.-S. Suppression of aging and plasticization in highly permeable polymers. Polymer 77, 377–386 (2015).

[b35] Son S.-Y. . One-step synthesis of carbon nanosheets converted from a polycyclic compound and their direct use as transparent electrodes of ITO-free organic solar cells. Nanoscale 6, 678–682 (2014). 24162657 10.1039/c3nr04828d

[b36] Kim B. G. . Sulfonation of PIM-1 towards highly oxygen permeable binders for fuel cell application. Macromol. Res. 22, 92–98 (2014).

[b37] Song J. . Linear high molecular weight ladder polymers by optimized polycondensation of tetrahydroxytetramethylspirobisindane and 1,4-dicyanotetrafluorobenzene. Macromolecules 41, 7411–7417 (2008).

[b38] Li P. . Characterization of carbon nanofiber composites synthesized by shaping process. Carbon 43, 2701–2710 (2005).

[b39] Sadezky A., Muckenhuber H., Grothe H., Niessner R. & Poschl U. Raman micro spectroscopy of soot and related carbonaceous materials: Spectral analysis and structural information. Carbon 43, 1731–1742 (2005).

[b40] Moon I. K., Lee J., Ruoff R. S. & Lee H. Reduced graphene oxide by chemical graphitization. Nat. Commun. 1, 73 (2010). 20865806 10.1038/ncomms1067

[b41] Li Z. . Carbonized chicken eggshell membranes with 3d architectures as high-performance electrode materials for supercapacitors. Adv. Energy Mater. 2, 431–437 (2012).

[b42] Xu H. X., Guo J. R. & Suslick K. S. Porous carbon spheres from energetic carbon precursors using ultrasonic spray pyrolysis. Adv. Mater. 24, 6028–6033 (2012). 22927232 10.1002/adma.201201915

[b43] Amato L. . Pyrolysed 3D-carbon scaffolds induce spontaneous differentiation of human neural stem cells and facilitate real-time dopamine detection. Adv. Funct. Mater. 24, 7042–7052 (2014).

[b44] Zhi L. . From well-defined carbon-rich precursors to monodisperse carbon particles with hierarchic structures. Adv. Mater. 19, 1849–1853 (2007).

[b45] Geise G. M., Park H. B., Sagle A. C., Freeman B. D. & McGrath J. E. Water permeability and water/salt selectivity tradeoff in polymers for desalination. J. Membr. Sci. 369, 130–138 (2011).

[b46] Geise G. M., Paul D. R. & Freeman B. D. Fundamental water and salt transport properties of polymeric materials. Prog Polym Sci 39, 1–42 (2014).

[b47] Striolo A. The mechanism of water diffusion in narrow carbon nanotubes. Nano Lett. 6, 633–639 (2006). 16608257 10.1021/nl052254u

[b48] Ma M., Tocci G., Michaelides A. & Aeppli G. Fast diffusion of water nanodroplets on graphene. Nat. Mater. 15, 66–72 (2016). 26480227 10.1038/nmat4449

[b49] Li Z. T. . Effect of airborne contaminants on the wettability of supported graphene and graphite. Nat. Mater. 12, 925–931 (2013). 23872731 10.1038/nmat3709

[b50] Sheng Y.-J., Jiang S. Y. & Tsao H.-K. Effects of geometrical characteristics of surface roughness on droplet wetting. J. Chem. Phys. 127, 234704 (2007). 18154406 10.1063/1.2804425

[b51] Huh C. & Mason S. G. Effects of surface-roughness on wetting (theoretical). J. Colloid Interf. Sci. 60, 11–38 (1977).

[b52] Van Wagner E. M., Sagle A. C., Sharma M. M. & Freeman B. D. Effect of crossflow testing conditions, including feed pH and continuous feed filtration, on commercial reverse osmosis membrane performance. J. Membr. Sci. 345, 97–109 (2009).

[b53] Walther J. H., Ritos K., Cruz-Chu E. R., Megaridis C. M. & Koumoutsakos P. Barriers to superfast water transport in carbon nanotube membranes. Nano Lett. 13, 1910–1914 (2013). 23521014 10.1021/nl304000k

[b54] Schaep J., Van der Bruggen B., Vandecasteele C. & Wilms D. Influence of ion size and charge in nanofiltration. Sep. Purif. Technol. 14, 155–162 (1998).

[b55] Fornasiero F. . Ion exclusion by sub-2-nm carbon nanotube pores. P. Natl. Acad. Sci. USA 105, 17250–17255 (2008). 10.1073/pnas.0710437105 PMC2582302 18539773

[b56] Kim D.-G., Kang H., Han S. & Lee J.-C. Dual effective organic/inorganic hybrid star-shaped polymer coatings on ultrafiltration membrane for bio- and oil-fouling resistance. ACS Appl. Mater. Interfaces 4, 5898–5906 (2012). 23054388 10.1021/am301538h

[b57] Kim H. J. . Polyphenol/Fe^III^ complex coated membranes having multifunctional properties prepared by a one-step fast assembly. Adv. Mater. Interfaces 2, 1500298 (2015).

[b58] Kim D.-G., Kang H., Choi Y.-S., Han S. & Lee J.-C. Photo-cross-linkable star-shaped polymers with poly(ethylene glycol) and renewable cardanol side groups: synthesis, characterization, and application to antifouling coatings for filtration membranes. Polym. Chem. 4, 5065–5073 (2013).

[b59] Sun X. H., Wu J., Chen Z. Q., Su X. & Hinds B. J. Fouling characteristics and electrochemical recovery of carbon nanotube membranes. Adv. Funct. Mater. 23, 1500–1506 (2013).

[b60] Kim D.-G., Kang H., Han S. & Lee J.-C. The increase of antifouling properties of ultrafiltration membrane coated by star-shaped polymers. J. Mater. Chem. 22, 8654–8661 (2012).

[b61] Kim D.-G., Kang H., Han S., Kim H. J. & Lee J.-C. Bio- and oil-fouling resistance of ultrafiltration membranes controlled by star-shaped block and random copolymer coatings. RSC Adv. 3, 18071–18081 (2013).

[b62] Banerjee I., Pangule R. C. & Kane R. S. Antifouling coatings: recent developments in the design of surfaces that prevent fouling by proteins, bacteria, and marine organisms. Adv. Mater. 23, 690–718 (2011). 20886559 10.1002/adma.201001215

[b63] Yang R., Jang H., Stocker R. & Gleason K. K. Synergistic prevention of biofouling in seawater desalination by zwitterionic surfaces and low-level chlorination. Adv. Mater. 26, 1711–1718 (2014). 24375685 10.1002/adma.201304386 PMC4041402

[b64] Mo Y. H. . Improved antifouling properties of polyamide nanofiltration membranes by reducing the density of surface carboxyl groups. Environ. Sci. Technol. 46, 13253–13261 (2012). 23205860 10.1021/es303673p

