# Supplementary material for: A Carbonaceous Membrane based on a Polymer of Intrinsic Microporosity (PIM-1) for Water Treatment
Source: Sci Rep. 2016 Oct 26;6:36078. doi: 10.1038/srep36078 (PMC5080592; doi:10.1038/srep36078)
Supplement: Supplementary Information [file srep36078-s1.doc]

Supplementary Information for the manuscript entitled:

**A Carbonaceous Membrane based on a Polymer of Intrinsic Microporosity (PIM-1) for Water Treatment**

Hee Joong Kim,^1^ Dong-Gyun Kim,^2,3^ Kyuchul Lee,^2,3^ Youngbin Baek,^1^ Youngjae Yoo,^2,3^ Yong Seok Kim,^2,3^ Byoung Gak Kim,^2,3^* and Jong-Chan Lee^1^*

^1^ School of Chemical and Biological Engineering and Institute of Chemical Processes, Seoul National University, 599 Gwanak-ro, Gwanak-gu, Seoul 08826, Republic of Korea

^2^ Advanced Materials Division, Korea Research Institute of Chemical Technology, 141 Gajeongro, Yuseong-gu, Daejeon 34114, Republic of Korea

^3^ Department of Chemical Convergence Materials, University of Science and Technology, 217 Gajeongro, Yuseong-gu, Daejeon 34114, Republic of Korea

*Corresponding Author

**Prof. J. –C. Lee**

Tel: +82-2-880-7070, Fax: +82-2-

E-mail: jongchan@snu.ac.kr

**Dr. B. G. Kim**

Tel: +82-42-861-4151, Fax: +82-42-860-7305

E-mail: bgkim@krict.re.kr

**Table of Contents**

Materials ................................................................................................................................... 3

Synthesis of PIM-1 ................................................................................................................... 3

Preparation of PIM-1, carbonized PIM-1 (C-PIM-1), and oxygen (O_2_) plasma-treated C-PIM-1 (PC-PIM-1) membranes ........................................................................................................ 4

Membrane filtration experiments ............................................................................................. 5

Instrumentation and characterization techniques ..................................................................... 6

Membrane performances of the membranes in this study ....................................................... 9

XPS and EA elemental composition of the membranes ........................................................ 11

Diffusion coefficient and hydrated radius of the ions ............................................................ 12

Comparison of the membrane performances prepared in this study and others ...............…. 13

Digital photograph of the PIM-1, C-PIM-1, and PC-PIM-1 membranes ............................... 14

TGA result of PIM-1 .............................................................................................................. 15

Raman spectra of C-PIM-1 membranes with different degree of carbonization ....................16

Surface SEM images of PIM-1 and C-PIM-1 membranes ..................................................... 17

AFM surface morphologies of PIM-1 and C-PIM-1 membranes .......................................... 18

N­_2_ adsorption and desorption isotherms of PIM-1 and C-PIM-1 membranes ....................... 19

*C*_m_^W,F^ values of PIM-1 and C-PIM-1 membranes .................................................................. 20

Contact angle values and wetting behaviors of the membranes ............................................. 21

Raman spectra of the C-PIM-1 and PC-PIM-1 membranes. .................................................. 22

Surface SEM images of PC-PIM-1 membrane ........................ ............................................. 23

AFM surface morphologies of PC-PIM-1 membrane ............................................................ 24

Surface zeta potential values of the membranes membranes ................................................. 25

Various salt rejection rates of the C-PIM-1 and PC-PIM-1 membranes ................................ 26

References for Supplementary Information ........................................................................... 27

**Methods**

*Materials*: 5,5′,6,6′-Tetrahydroxy-3,3,3′,3′-tetramethyl-1,1′-spirobisindane (TTSBI, >97%, TCI Chemicals) was purified by recrystallization from a mixture of dichloromethane and methanol. Tetrafluoroterephthalonitrile (TFTPN, >98%, Matrix Scientific) was purified by vacuum sublimation at 150 °C under inert atmosphere. Potassium carbonate (K_2_CO_3_, 99.99%, anhydrous), bovine serum albumin (BSA, >96%), magnesium sulfate (MgSO_4_, >99.5%, anhydrous), sodium chloride (NaCl, >99%, anhydrous), magnesium chloride (MgCl_2_, >98%, anhydrous), sodium sulfate (Na_2_SO_4_, >99%, anhydrous), all from Sigma-Aldrich, were used as received. Deionized (DI) water with a resistivity of 18.3 mΩ cm ]was obtained from a water purification system (Synergy, Millipore, USA). A commercial nanofiltration (NF) membrane, NF2A, was purchased from Sepro Membranes (Oceanside, CA, USA). All other reagents and solvents, such as methanol (MeOH), dimethylformamide (DMF), tetrahydrofuran (THF), and chloroform (CHCl_3_), were used as received from standard suppliers.

*Synthesis of PIM-1*: PIM-1 was synthesized according to a previously reported procedure with slight modifications.^1-4^ Under nitrogen (N_2_) flow, TTSBI (3.4 g, 10 mmol), K_2_CO_3_ (4.1 g, 30 mmol), TFTPN (2.0 g, 10 mmol), and DMF (70 mL) were added to a 250 mL two-necked round-bottomed flask equipped with a condenser. The reaction mixture was stirred at 55 °C for 72 h. After polymerization, THF (~140 mL) was added into the flask for removing low-molecular-weight oligomers. The resulting solution was precipitated into an excess of water. The polymer so obtained was dissolved in THF, and the solution was precipitated into MeOH. After drying under vacuum for several days, a yellow solid product was obtained in 75% yield. The chemical structure of PIM-1 was confirmed by ^1^H-NMR (Bruker Advance 700 MHz spectrometer) using tetramethylsilane (TMS) as the reference; ^1^H-NMR (700 MHz, CDCl_3_, *δ*): 1.307 and 1.365 (−C*H*_3_), 2.159 and 2.328 (−C*H*_2_−), and 6.415 and 6.806 (C*H*, aromatic). Anal. calcd for C_29_H_20_N_2_O_4_: C 75.64, H 4.38, N 6.08, O 13.90. Found: C 76.38, H 4.61, N 5.82, O 13.19. The molecular weight (*M*_n_) and molecular weight distribution (*Đ*) of PIM-1 were determined by gel-permeation chromatography (GPC); *M*_n_ = 50,100 g mol^−1^, *Đ* = 1.87.

*Preparation of PIM-1, carbonized PIM-1 (C-PIM-1), and oxygen (O_2_) plasma-treated C-PIM-1 (PC-PIM-1) membranes*: PIM-1 membrane was prepared by a simple solution casting method. First, the PIM-1 was dissolved in CHCl_3_ (0.5−2.0 wt%), and the solution was poured into a glass dish (diameter = 10 cm). After drying this solution at ambient temperature for 2 days, followed by drying under vacuum at 60 °C overnight, a free-standing, transparent, and light yellow PIM-1 membrane was obtained. The thickness of the PIM-1 membrane was controlled by changing the concentration and amount of polymer solution, e.g., for preparing a 30 µm thick PIM-1 membrane, a 1.0 wt%, 16.5 mL PIM-1 solution was utilized. The thickness was measured using a micrometer (Kett LZ-370, Agelec Enterprises Pty Ltd).

The C-PIM-1 membrane was prepared by the controlled thermal treatment of the PIM-1 membrane. The PIM-1 membrane was placed on a silicon wafer (5 cm × 5 cm) in a furnace (Nabertherm P330) under N_2_/H_2_ atmosphere (100 cc min^−1^, 95/5 vol%). The membrane was carefully pressed using another silicon wafer (5 cm × 5 cm) for preventing the deformation of membrane during thermal treatment. The following procedure was used for the preparation of C-PIM-1 membranes: First, the PIM-1 membrane was placed in a furnace (Nabertherm P330) and vacuum was applied. After the furnace was refilled with N_2_/H_2_ gas (95/5 vol%), flow rate of the mixed gas was controlled to be 100 mL min^−1^. Then, the furnace was heated to a specific temperature (1100-1300 ^o^C) at 5 ^o^C min^−1^. The membrane was kept at the final temperature for a specific time range (1-6 h). The degree of carbonization was controlled by changing the final temperature and dwell time; for example, PIM-1 membranes were carbonized at 1100 ^o^C for 2 h, at 1200 ^o^C for 3 h, and 1300 ^o^C for 6 h to obtain the C-PIM-1 membranes with a degree of carbonization of 40%, 50%, and 60%, respectively. The degree of carbonization is defined as membrane weight loss (%) during thermal treatment.

Both surfaces of the C-PIM-1 membrane were subjected to O_2_ plasma treatment for preparing the PC-PIM-1 membrane. The plasma treatment instrument consisting of parallel electrodes operated at a radio-frequency of 13.56 MHz. The C-PIM-1 membrane was placed on a powered electrode under an Ar/O_2_ flow (71/29 vol% and 30 sccm). Time and power of O_2_ plasma treatment for preparing the PC-PIM-1 membrane were 30 s and 185 W, respectively. Distinct performance changes were not observed when the time and power of O_2_ plasma treatment were changed in the range from 10 to 300 s and from 50 to 185 W, respectively.

*Membrane filtration experiments*: Membrane filtration for pure water and an aqueous MgSO_4_ solution (2,000 ppm) was conducted using a stirred dead-end filtration cell (CF042, Sterlitech Corp., Kent, WA). The feed side of the system was subjected to 10 bar pressure by N_2_, and all experiments were conducted at an agitation speed of 200 rpm and room temperature. The water flux (*J*) measured by weighing the permeate solution at a given time was calculated as follows:

*J* = *ΔV* / (*A* × *Δ*t) (Eq. 1)

where *∆V* is the volume of permeate collected between two weight measurements, *A* is the membrane surface area, and *∆t* is the time between two weight measurements. The salt rejection rate (*R*) was calculated as follows:

*R* = (1 − *C*_p_ / *C*_f_) × 100 % (Eq. 2)

where *C*_p_ and *C*_f_ represent the salt concentrations in the permeate and feed, respectively. Salt concentrations were measured using a conductivity meter (InoLab Cond 730P, WTW 82362, Weilheim). To investigate the Donnan exclusion ability of the membrane, solutions of various salts with different ion valences (Na_2_SO_4_, MgSO_4_, NaCl, and MgCl_2_) were used (Fig. S10). As the salt rejection mechanism of NF membranes is normally explained in terms of charge, size effect (sieving), and/or diffusion of salts, filtration experiments were conducted with a relatively low feed pressure (5 bar) and low salt concentration (10 mM) for minimizing the transport of ions by convection and diffusion, respectively.^5,6^ The water flux and salt rejection values shown in this study are the average values obtained by three re-test measurements from more than two membrane samples.

For the fouling resistance test, a BSA aqueous solution (1 g L^−1^) was forced to permeate through the membranes, and the water flux was recorded at each time. The initial water flux values of the membranes were controlled to reach 55 LMH by controlling the feed pressure. The flux decline ratio (DR) was calculated as follows:

DR = (1 – *J*_250_ / *J*_0_) × 100 % (Eq. 3)

where *J*_0_ and *J*_250_ represent the initial water flux and the water flux recorded at 250 min after the filtration of the initial feed, respectively.

*Instrumentation and characterization techniques*: The chemical structure and molecular weight of PIM-1 were confirmed by ^1^H-NMR (Bruker Advance 700 MHz spectrometer) and gel-permeation chromatography (GPC, Wyatt Technology), respectively. Thermogravimetric analysis (TGA) was performed using a Pyris 1 TGA apparatus from PerkinElmer at a heating rate of 10 °C min^–1^ under N_2_. The membrane surface composition was investigated by X-ray photoelectron spectroscopy (XPS, PHI-1600) using Mg Kα (1254.0 eV) as the radiation source. Survey spectra were collected in the range of 0–1100 eV, followed by the high-resolution scan of the C 1s, O 1s, and N 1s regions. The membrane atomic composition was analyzed using an elemental analyzer (EA, Flash2000, Thermo Scientific). Raman spectra were recorded on a LabRam Aramis Raman spectrometer (Horiba Jobin-Yvon). The excitation source was a diode laser with an excitation wavelength of 785 nm and a power of 5 mW. The Raman scattered light signal was collected in a back-scattering geometry using a 100× microscope objective lens. The diameter of the Raman excitation beam spot was approximately 1 µm. Nitrogen adsorption isotherms were measured by surface characterization analyzer (3flex 3500, Micromeritics) at 77 K. The membranes were degassed at 200 °C for 24 h before the measurements. The spectific surface area and median pore size of the membranes were caculated by using the Brunauer-Emmett-Teller (BET) method and Horvath-Kawazoe model, respectively. The membrane surface morphology was investigated by scanning electron microscopy (SEM, SigmaHD, Carl Zeiss) and atomic force microscopy (AFM, Asylum Research MFP-3D). The wetting behaviors of water droplets on the membranes were evaluated using a contact angle analyzer (KRÜSS DSA100) by the sessile drop method. Three replicate measurements were conducted for each membrane. The PC-PIM-1 membrane was prepared by the O_2_ plasma treatment of the C-PIM-1 membrane using a plasma reactor (Korea Vacuum Co.). Zeta potential values of the membrane surfaces were recorded on an electrophoretic light scattering spectrophotometer (ELS-8000, Otsuka Electronics Co.). The membrane water uptake was determined by measuring the weight before and after membranes (1 cm × 1 cm) were soaked in deionized water for 24 h. After the membrane was removed and wiped, the weight of the wet membrane was obtained; it was calculated as follows:

Water uptake (%) = [(*W*_wet_ – *W*_dry_) / *W*_dry_] ×100 (Eq. 4)

where *W*_wet_ and *W*_dry_ represent the weights of the wet and dry membranes, respectively. The equilibrium water concentration in the membrane (*C*^m^_W,F_) was obtained from the water uptake experiments, which was caculated as follows:

*C*^m^_W,F_ = (*W*_wet_ – *W*_dry_) / *V*_dry_  (Eq. 5)

where *V*_dry_ is the volume of the dry membrane.

**Table S1.** Pure water flux (PWP), water flux (WF), and salt rejection (R) values of the membranes in this study. The water flux and salt rejection values were obtained by filtration of MgSO_4_ solution (2,000 ppm).

| Membrane | Thickness  (µm) | Carbonization  (%) | PWP  (LMH bar^–1^) | WF  (LMH bar^–1^) | R  (%) |
| --- | --- | --- | --- | --- | --- |
| NF2A | - | - | 4.66 ± 0.20 | 3.32 ± 0.11 | 76.86 ± 1.59 |
| PIM-1 | 20 | - | 0.31 ± 0.01 | 0.13 ± 0.00 | 91.18 ± 0.18 |
|  | 30 |  | 0.23 ± 0.02 | 0.12 ± 0.00 | 91.41 ± 0.23 |
|  | 37.5 |  | 0.18 ± 0.03 | 0.09 ± 0.01 | 91.78 ± 0.15 |
|  | 40 |  | 0.13 ± 0.00 | 0.09 ± 0.00 | 91.42 ± 0.33 |
|  | 50 |  | 0.12 ± 0.01 | 0.09 ± 0.00 | 92.16 ± 0.29 |
|  | 60 |  | 0.11 ± 0.01 | 0.09 ± 0.01 | 93.39 ± 0.17 |
|  | 70 |  | 0.10 ± 0.00 | 0.09 ± 0.00 | 93.69 ± 0.21 |
| C-PIM-1 | 30 | 40 | 4.85 ± 0.17 | 3.51 ± 0.16 | 82.69 ± 1.91 |
|  |  | 47.5 | 5.32 ± 0.05 | 3.73 ± 0.05 | 82.94 ± 0.68 |
|  |  | 50 | 5.65 ± 0.19 | 3.90 ± 0.05 | 81.54 ± 0.59 |
|  |  | 60 | 6.43 ± 0.09 | 4.45 ± 0.02 | 78.76 ± 1.21 |
|  | 20 | 37.5 | 7.08 ± 0.24 | 4.91 ± 0.05 | 79.29 ± 0.58 |
|  | 30 |  | 4.80 ± 0.17 | 3.30 ± 0.07 | 83.40 ± 0.35 |
|  | 35 |  | 4.09 ± 0.03 | 2.76 ± 0.09 | 83.64 ± 0.45 |
|  | 40 |  | 3.47 ± 0.04 | 2.38 ± 0.11 | 83.64 ± 0.53 |
|  | 45 |  | 3.12 ± 0.03 | 2.20 ± 0.07 | 79.29 ± 0.58 |
|  | 50 |  | 2.88 ± 0.02 | 1.96 ± 0.01 | 84.70 ± 0.04 |
|  | 55 |  | 2.64 ± 0.09 | 1.82 ± 0.01 | 84.65 ± 0.24 |
|  | 70 |  | 2.06 ± 0.06 | 1.47 ± 0.01 | 85.76 ± 0.16 |
|  | 20 | 60 | 10.57 ± 0.42 | 7.74 ± 0.18 | 78.58 ± 0.46 |

**Table S1.** (Continued)

| Membrane | Thickness  (µm) | Carbonization  (%) | PWP  (LMH bar^–1^) | WF  (LMH bar^–1^) | R  (%) |
| --- | --- | --- | --- | --- | --- |
| PC-PIM-1 | 30 | 40 | 7.04 ± 0.08 | 5.74 ± 0.10 | 79.60 ± 0.82 |
|  |  | 47.5 | 8.38 ± 0.07 | 6.33 ± 0.03 | 78.94 ± 0.52 |
|  |  | 50 | 8.82 ± 0.14 | 6.54 ± 0.02 | 77.38 ± 0.24 |
|  |  | 60 | 10.79 ± 0.24 | 7.95 ± 0.01 | 76.51 ± 1.18 |
|  | 20 | 37.5 | 10.40 ± 0.26 | 7.36 ± 0.13 | 75.91 ± 0.62 |
|  | 30 |  | 6.98± 0.11 | 4.91 ± 0.05 | 78.50 ± 0.41 |
|  | 35 |  | 5.92 ± 0.04 | 4.22 ± 0.07 | 80.94 ± 0.17 |
|  | 40 |  | 5.08 ± 0.04 | 3.71 ± 0.07 | 83.03 ± 0.19 |
|  | 45 |  | 4.55 ± 0.09 | 3.35 ± 0.02 | 83.55 ± 0.29 |
|  | 50 |  | 4.19 ± 0.04 | 3.07 ± 0.05 | 83.85 ± 0.26 |
|  | 55 |  | 3.80 ± 0.05 | 2.76 ± 0.06 | 84.03 ± 0.19 |
|  | 70 |  | 2.95 ± 0.05 | 2.21 ± 0.05 | 85.20 ± 0.51 |
|  | 20 | 60 | 15.43 ± 0.45 | 13.30 ± 0.57 | 77.37 ± 0.36 |

**Table S2.** Bulk and surface elemental compositions (at%) of PIM-1, C-PIM-1, and PC-PIM-1 (40% carbonization) membranes obtained by EA and XPS, respectively.

| Membrane | Bulk Composition (EA) | | | Surface Composition (XPS) | | |
| --- | --- | --- | --- | --- | --- | --- |
|  | C | O | N | C | O | N |
| PIM-1 | 83.69 | 10.85 | 5.46 | 82.60 | 13.60 | 3.70 |
| C-PIM-1 | 98.22 | 1.09 | 0.69 | 96.82 | 1.37 | 1.81 |
| PC-PIM-1 | 98.14 | 1.16 | 0.70 | 87.51 | 11.60 | 0.89 |

**Table S3.** Diffusion coefficient values (*D*_i_) of salts (at 25 ^o^C) and hydrated ionic radius (*R*_i_) of the corresponding ions.^5,6^

| Salt | *D*_i_ (× 10^9^, m^2^ s^–1^) | Ion | *R*_i_ (nm) |
| --- | --- | --- | --- |
| NaCl | 1.61 | Cl^–^ | 0.33 |
| Na_2_SO_4_ | 1.23 | Na^+^ | 0.36 |
| MgCl_2_ | 1.25 | SO_4_^2–^ | 0.38 |
| MgSO_4_ | 0.85 | Mg^2+^ | 0.44 |

**Table S4.** MgSO_4_ rejection (R) and water flux (WF) values of optimized C-PIM-1 and PC-PIM-1 membranes in this study and other NF membranes in the literature.

| Name | Membrane  Type | Selective Layer | WF  (LMH bar^–1^) | R (%) | MgSO_4_ Concentration | Ref |
| --- | --- | --- | --- | --- | --- | --- |
| NF2A | TFC*^a^* | Polyamide | 3.32 | 76.86 | 2,000 ppm | This  Work |
| C-PIM-1*^c^* | Single*^b^* |  |  |  |  |  |
| PC-PIM-1*^c^* |  |  |  |  |  |  |
| GNm | Single | Graphene | 4.76 | 82.8 | 1,200 ppm | 7 |
| G-CNTm (8:1) |  | Graphene-CNT | 8.02 | 44.2 |  |  |
| G-CNTm (4:1) |  |  | 8.05 | 42.3 |  |  |
| G-CNTm (8:3) |  |  | 9.51 | 40.6 |  |  |
| G-CNTm (2:1) |  |  | 11.33 | 30.9 |  |  |
| G-CNTm (8:5) |  |  | 12.13 | 25.1 |  |  |
| uGNM | Single | Graphene | 3.3 | 30.0 | 2,400 ppm | 8 |
| Cub_bi_ | Single | Cubic liquid crystal | 3.73 | 26.0 | 1,500 ppm | 9 |
| Amorphous | Single | liquid crystal | 8.40 | 20.0 |  |  |
| TFC-NFM | TFC | Zirconia | 8.33 | 69.0 | 2,000 ppm | 10 |
| TMC | TFC | Polyamide | 6.20 | 94.5 | 3,000 ppm | 11 |
| IPC |  |  | 2.90 | 77.7 |  |  |

*^a^*Thin-film composite membrane. *^b^*Single layer membrane. *^c^*20 µm thickness, 60% carbonization.


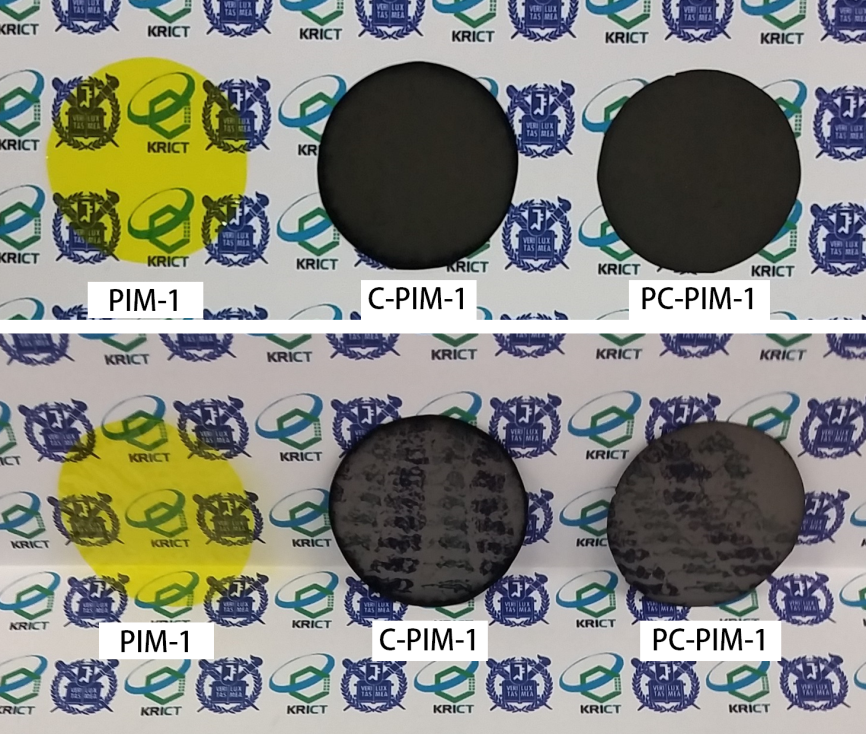


**Figure S1.** Photographs of PIM-1, C-PIM-1, and PC-PIM-1 (40% carbonization) membranes. The membranes in the upper row are laid on a desk, whereas those in bottom row stand against the wall to show the glittering surfaces after carbonization.

**
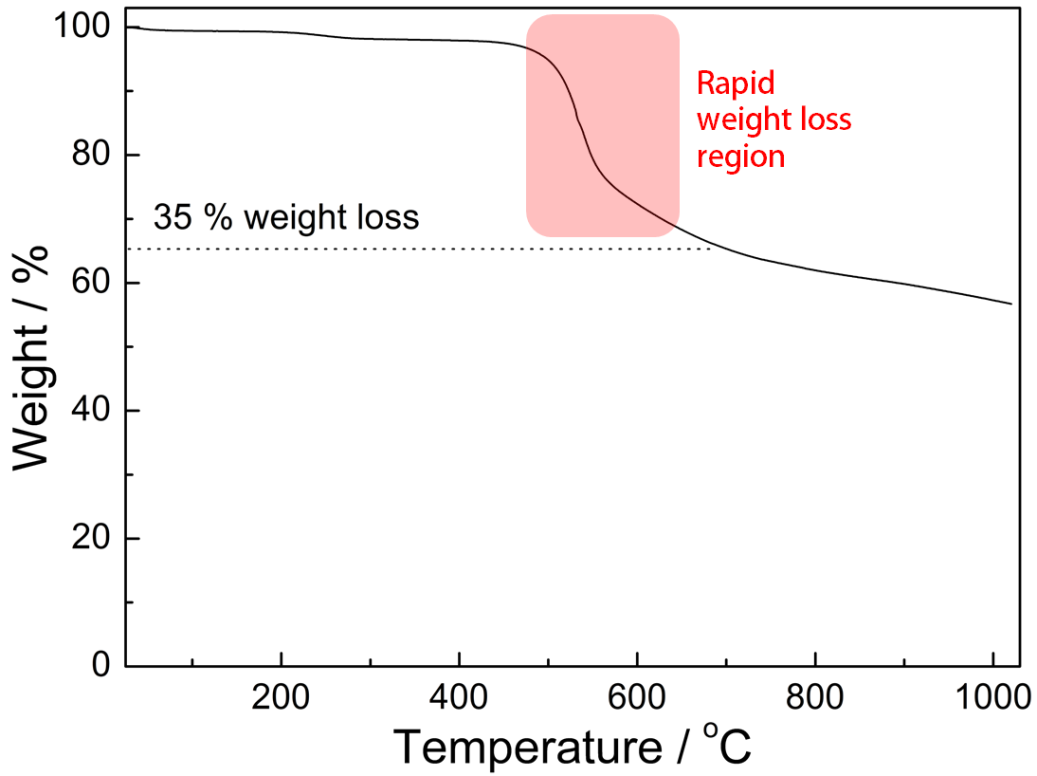
**

**Figure S2.** TGA curve of PIM-1.


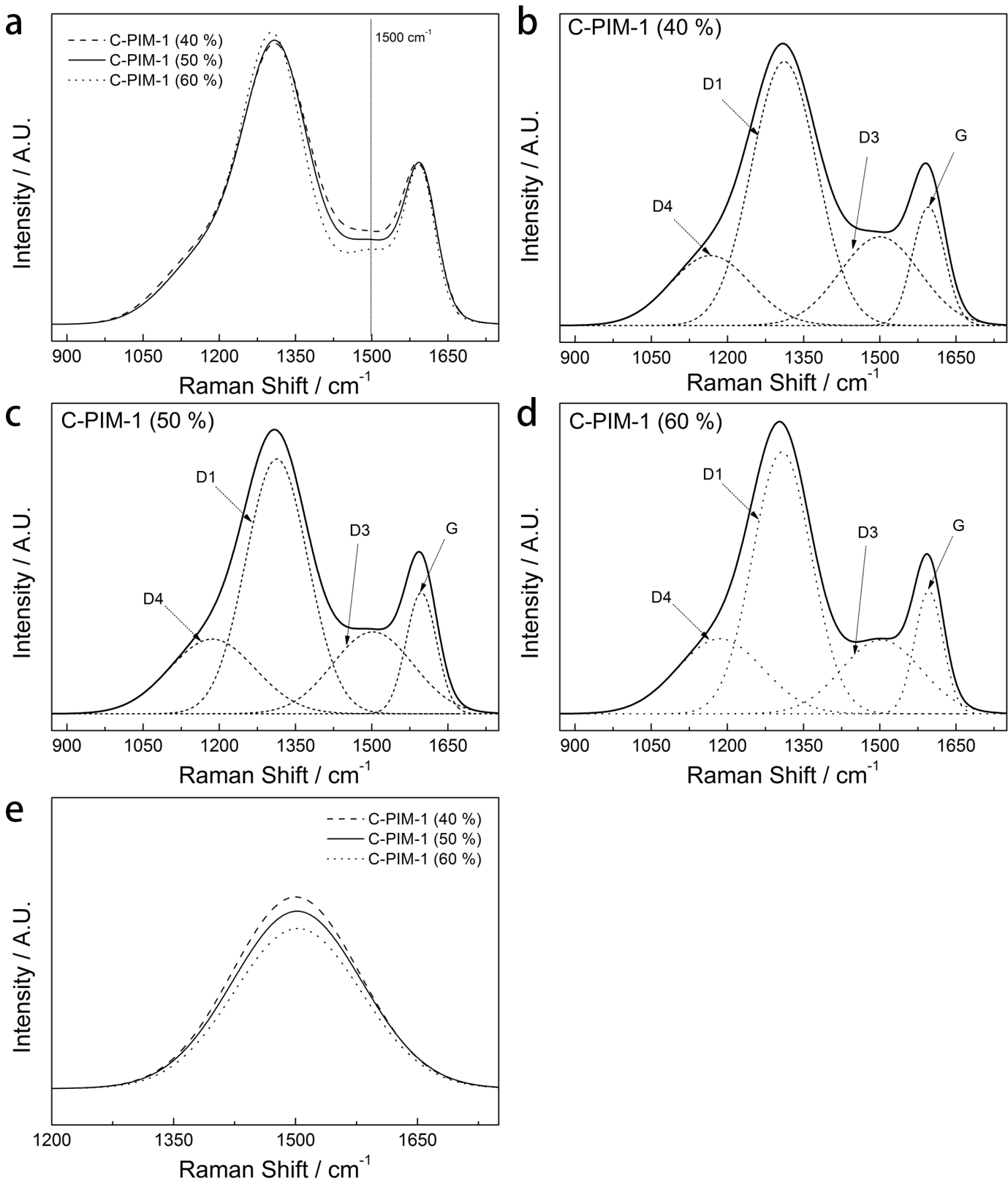


**Figure S3.** Raman spectra of C-PIM-1 membranes. G band peak intensities in all the Raman spectra were normalized for clear comparison. a) Raman spectra of C-PIM-1 membranes with different degrees of carbonization. Deconvoluted Raman spectra of C-PIM-1 membranes with b) 40%, c) 50%, and d) 60% carbonization using Gaussian curve fitting for the D1, D3, D4, and G band peaks. e) D3 band peaks of C-PIM-1 membranes with different degrees of carbonization.

**
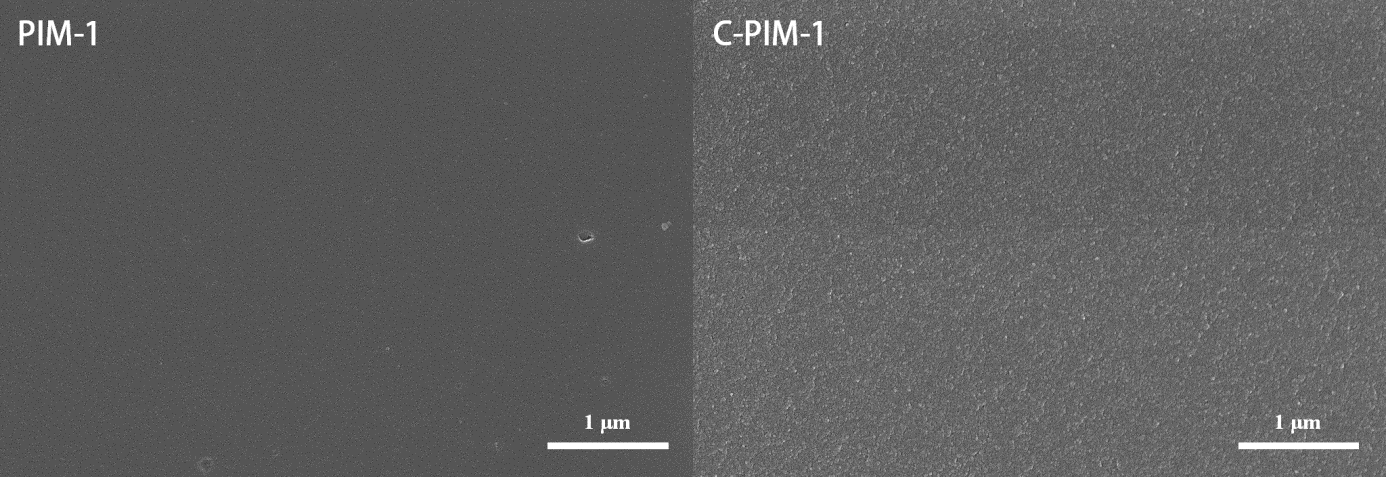
**

**Figure S4.** SEM images of PIM-1 and C-PIM-1 (40% carbonization) membrane surfaces.


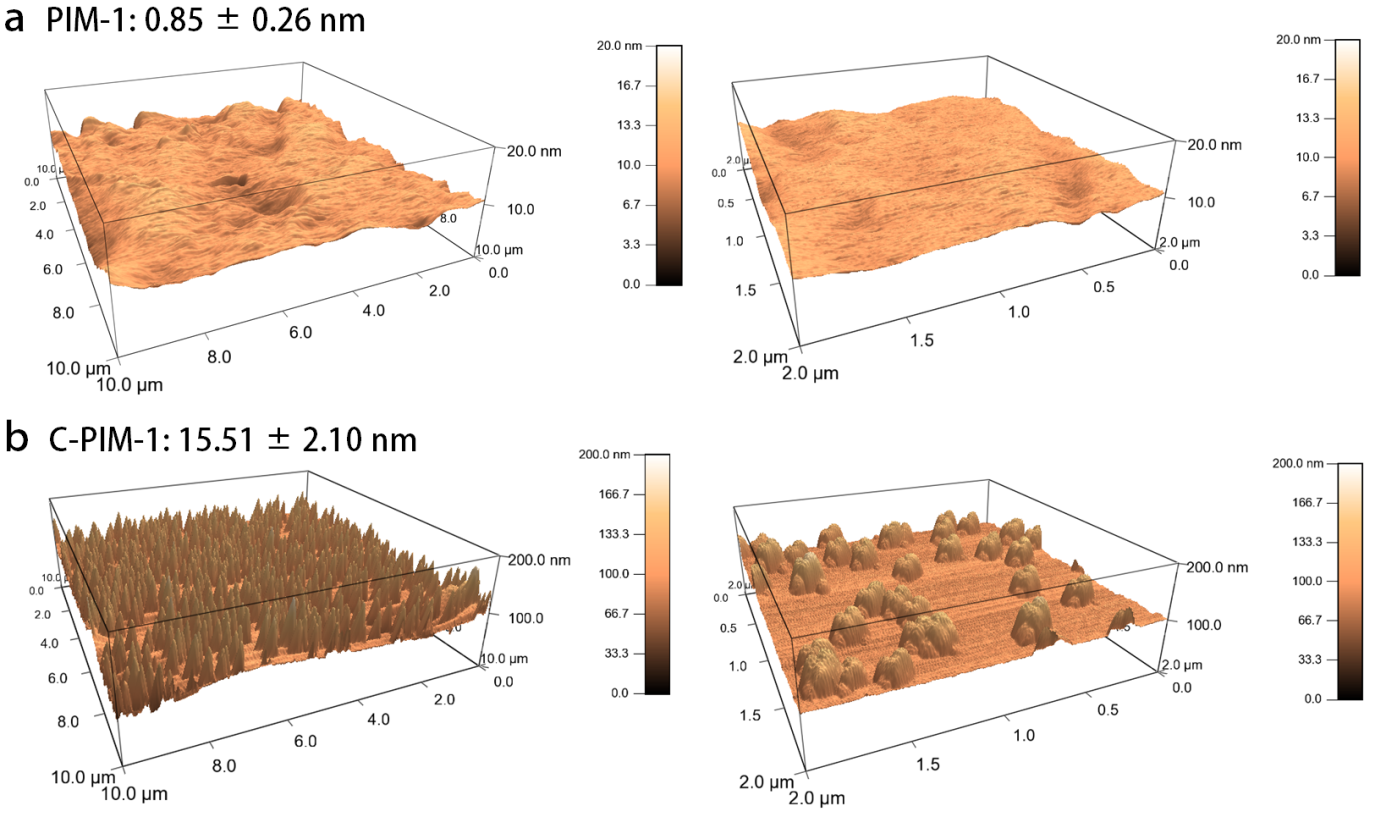


**Figure S5**. AFM surface morphologies and root mean square (RMS) roughness values of a) PIM-1 and b) C-PIM-1 (40% carbonization) membranes (left, 10 µm × 10 µm; right, 2 µm × 2 µm images).

**
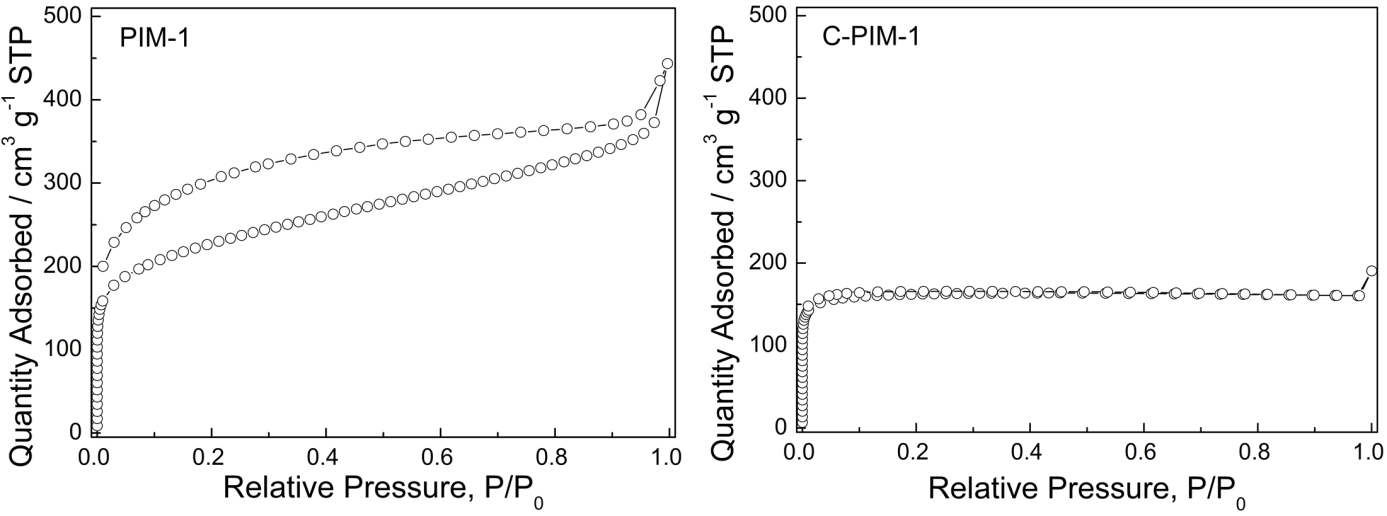
**

**Figure S6.** N_2_ adsorption and desorption isotherms of PIM-1 and C-PIM-1 (40% carbonization) at 77 K.


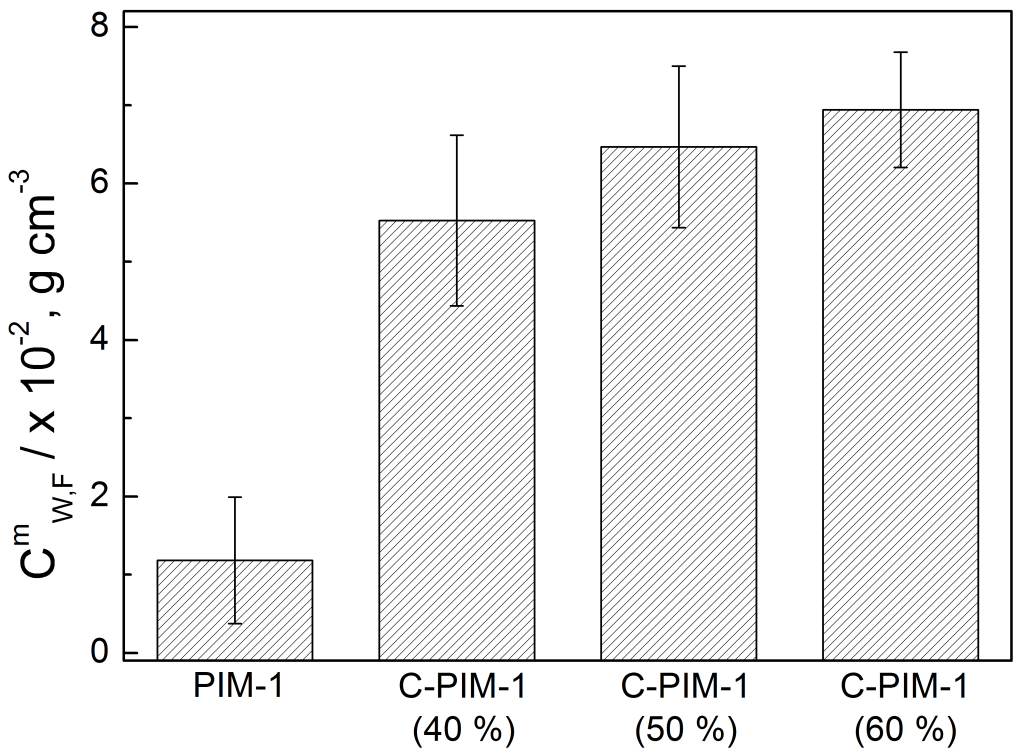


**Figure S7.** *C*^m^_W,F_ values of PIM-1 and C-PIM-1 membranes (carbonization = 40–60%) obtained from water uptake measurements.


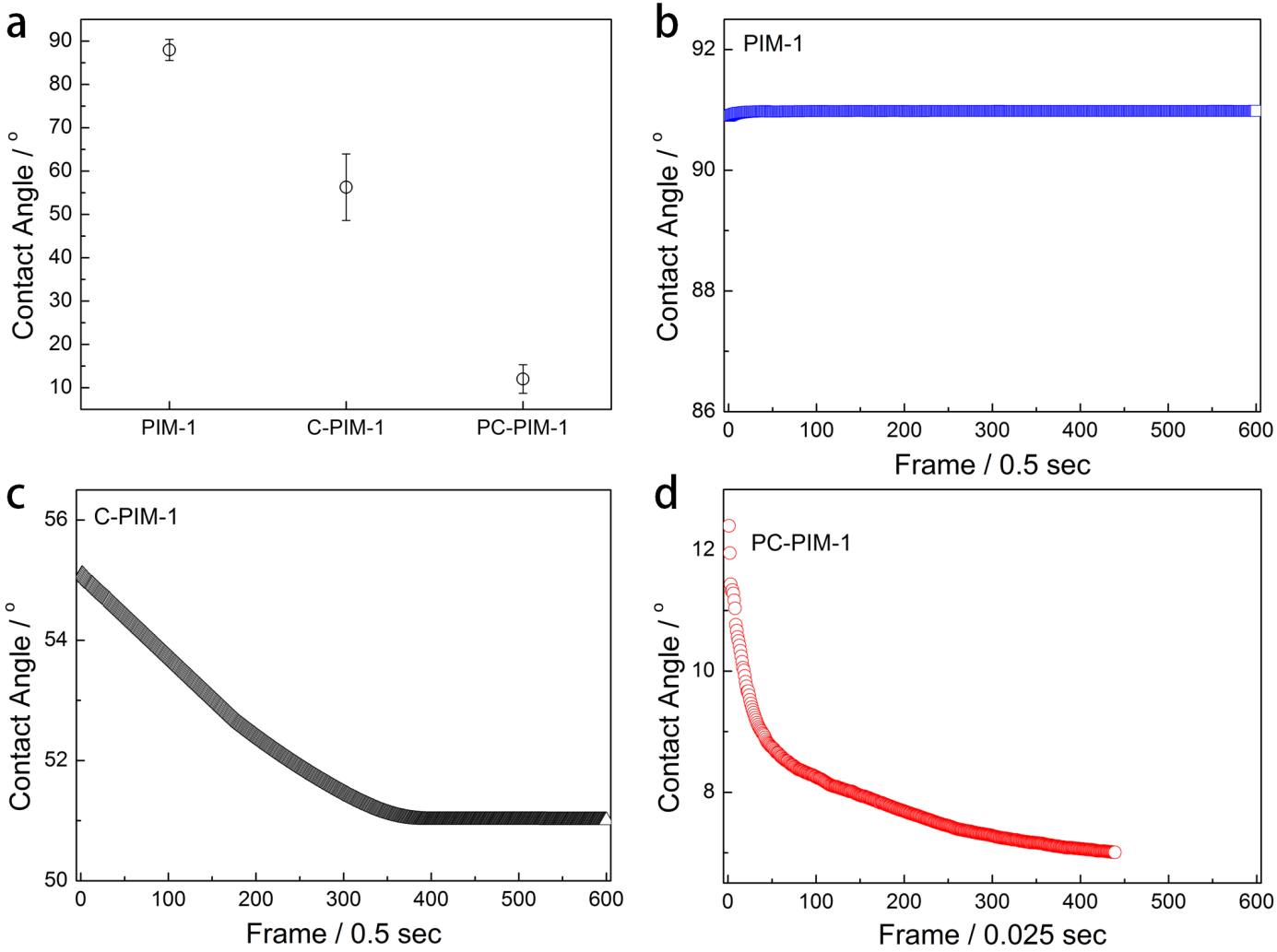


**Figure S8.** a) Sessile drop water contact angle values of PIM-1, C-PIM-1, and PC-PIM-1 membranes. Wetting behaviors of water droplets on b) PIM-1, c) C-PIM-1 (40% carbonization), and d) PC-PIM-1 (40% carbonization) membranes. The relative humidity was maintained over 65% to minimize the evaporation of water droplets during the wetting experiment. Much shorter frame time (1 frame = 0.025 sec), compared to that for PIM-1 and C-PIM-1 membranes (1 frame = 0.5 sec), was used for the PC-PIM-1 membrane due to the rapid decrease of water contact angle on the membrane surface.

`


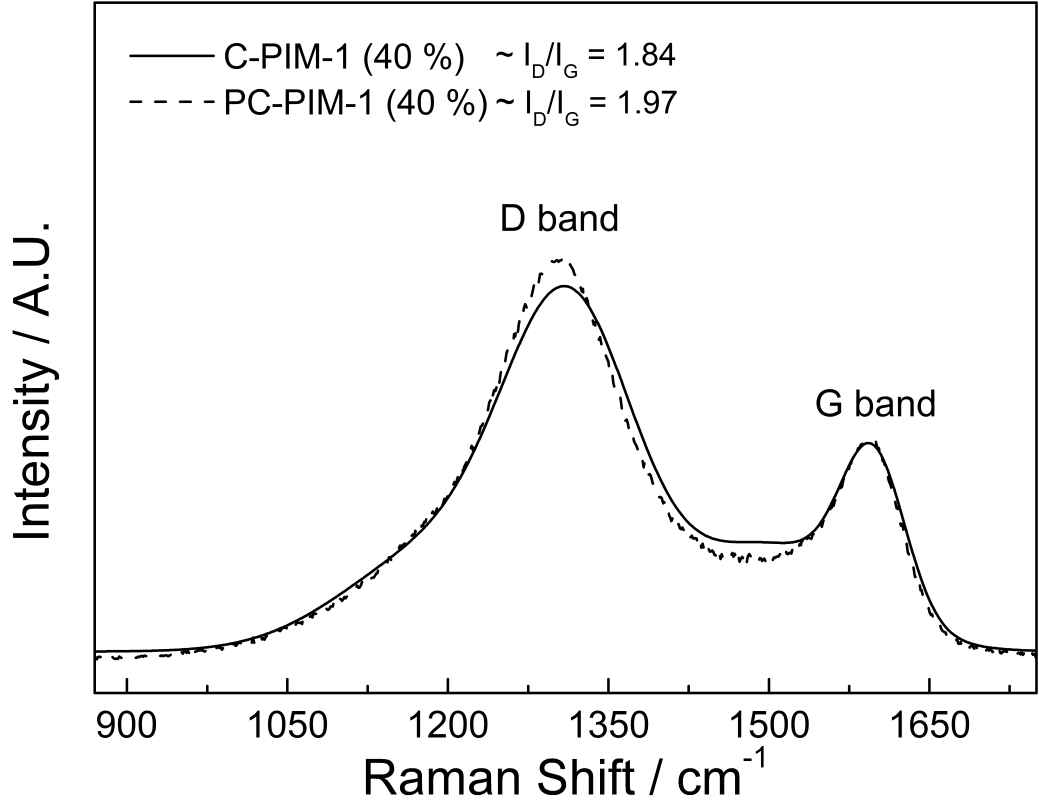


**Figure S9.** Raman spectra of C-PIM-1 and PC-PIM-1 membranes with 40% carbonization. The G band peak intensities were normalized for clear comparison.


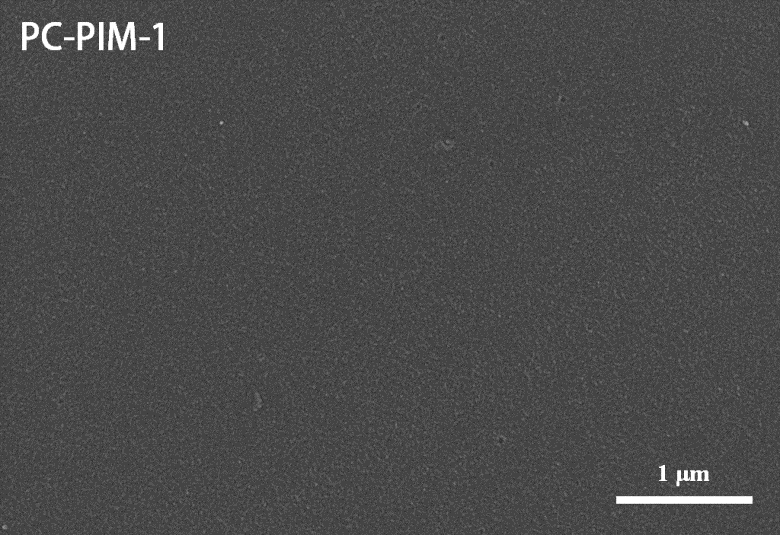


**Figure S10.** SEM images of the PC-PIM-1 (40% carbonization) membrane surface.

**
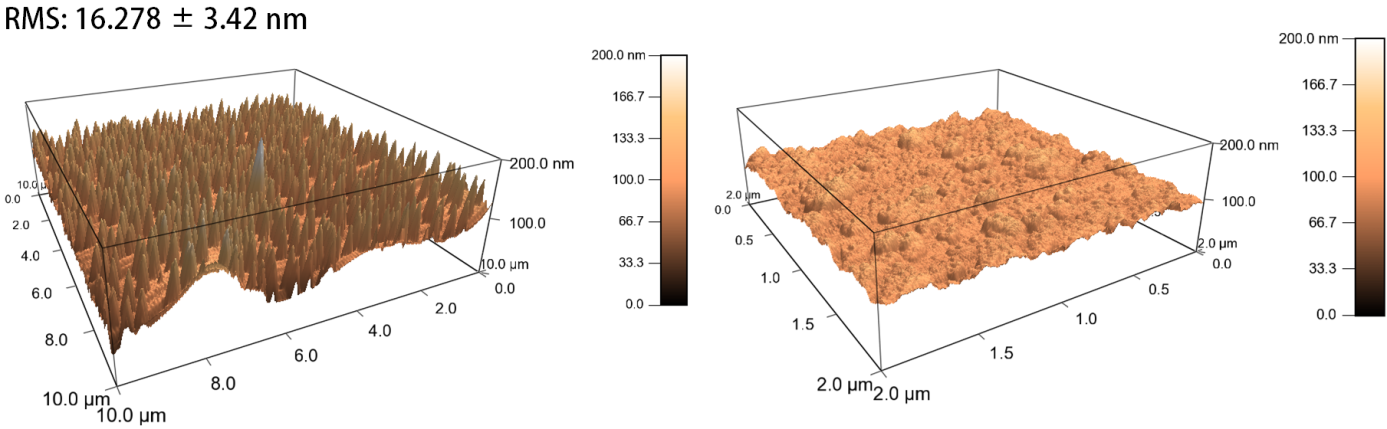
**

**Figure S11.** AFM surface morphologies and root mean square (RMS) roughness value of the PC-PIM-1 membrane (40% carbonization) (left, 10 µm × 10 µm; right, 2 µm × 2 µm images).


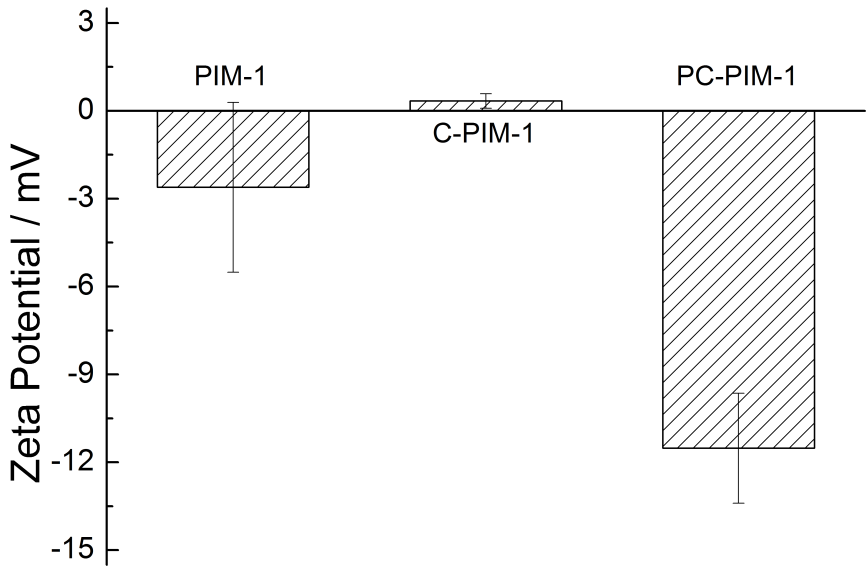


**Figure S12.** Zeta potential values of PIM-1, C-PIM-1 (40% carbonization), and PC-PIM-1 (40% carbonization) membranes.


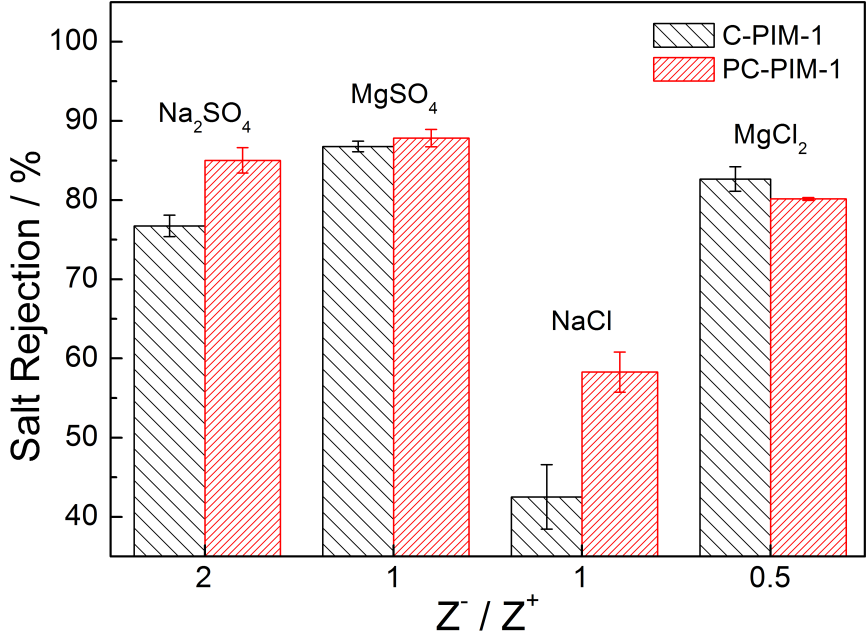


**Figure S13.** Salt rejection rates of C-PIM-1 and PC-PIM-1 membranes (40% carbonization) for various salt solutions (10 mM). Filtrations were conducted under 5 bar of feed pressure with a stirring speed of 200 rpm.

**References for supplementary information**

1. Kim, B. G. *et al*. Sulfonation of PIM-1 towards highly oxygen permeable binders for fuel cell application. *Macromol. Res.* **22**, 92-98 (2014).

2. Son, S. –Y. *et al.* One-step synthesis of carbon nanosheets converted from a polycyclic compound and their direct use as transparent electrodes of ITO-free organic solar cells. *Nanoscale* **6**, 678-682 (2014).

3. Budd, P. M. *et al.* Solution-processed, organophilic membrane derived from a polymer of intrinsic microporosity. *Adv. Mater.* **16**, 456-459 (2004).

4. Song, J. *et al.* Linear high molecular weight ladder polymers by optimized polycondensation of tetrahydroxytetramethylspirobisindane and 1,4-dicyanotetrafluorobenzene. *Macromolecules* **41**, 7411-7417 (2008).

5. Fornasiero, F. *et al.* Ion exclusion by sub-2-nm carbon nanotube pores. *P. Natl. Acad. Sci. USA* **105**, 17250-17255 (2008).

6. Schaep, J., Van der Bruggen, B., Vandecasteele, C. & Wilms, D. Influence of ion size and charge in nanofiltration. *Sep. Purif. Technol.* **14**, 155-162 (1998).

7. Han, Y., Jiang, Y. Q. & Gao, C. High-flux graphene oxide nanofiltration membrane intercalated by carbon nanotubes. *ACS Appl. Mater. Interfaces* **7**, 8147-8155 (2015).

8. Han, Y., Xu, Z. & Gao C. Ultrathin graphene nanofiltration membrane for water purification. *Adv. Funct. Mater.* **23**, 3693-3700 (2013).

9. Henmi, M. *et al.* Self-organized liquid-crystalline nanostructured membranes for water treatment: selective permeation of ions. *Adv. Mater.* **24**, 2238-2241 (2012).

10. Lv, Y., Yang, H. C., Liang, H. Q., Wan, L. S. & Xu, Z. K. Novel nanofiltration membrane with ultrathin zirconia film as selective layer. *J. Membr. Sci.* **500**, 265-271 (2016).

11. Mo, Y. H. *et al*. Improved antifouling properties of polyamide nanofiltration membranes by reducing the density of surface carboxyl groups. *Environ. Sci. Technol.* **46**, 13253-13261 (2012).
